# Supplementary material for: Deletion of wfs1 Impairs Oligodendrocyte Precursor Cells Dorsal Distribution and Myelination Through the wfs1-hmgcs1 Axis in Zebrafish
Source: Biology (Basel). 2026 Aug 16;15(16):1402. doi: 10.3390/biology15161402 (PMC13510151; doi:10.3390/biology15161402)
Supplement: Supplementary file 1 [file biology-15-01402-s001.zip › File S1-Transcriptome sequencing data and differentially expressed genes (DEGs) in wfs1-- zebrafish at 5 dpf/2.KEGG/wfs1KOvsWT/DOWN/src/dre00564.html]

dre00564


- dre:767688

- Down regulated genes

767688(-1.20360432923595)

- dre:406370

- Down regulated genes

406370\_1(-1.88308518916857)
- dre:767688

- Down regulated genes

767688(-1.20360432923595)
- dre:100148818

- Down regulated genes

100148818(-1.62621488580639)

- dre:724010

- Down regulated genes

724010(-6.84228486467569)

- dre:767688

- Down regulated genes

767688(-1.20360432923595)

- dre:406370

- Down regulated genes

406370\_1(-1.88308518916857)
- dre:767688

- Down regulated genes

767688(-1.20360432923595)
- dre:100148818

- Down regulated genes

100148818(-1.62621488580639)

- dre:767688

- Down regulated genes

767688(-1.20360432923595)

Close
